# Whole-transcriptome sequencing analysis reveal mechanisms of Yiqi Huoxue Yangyin (YHY) decoction in ameliorating D-gal-induced cardiac aging

**DOI:** 10.18632/aging.204532

**Published:** 2023-04-17

**Authors:** Xue Wang, Jiaqi Zhang, Chengkui Xiu, Jing Yang, Yiqing Liu, Yan Lei

**Affiliations:** 1 Beijing Key Laboratory of Research of Chinese Medicine on Prevention and Treatment for Major Diseases, Experimental Research Center, China Academy of Chinese Medical Sciences, Beijing 100700, China; 2 Xiyuan Hospital, China Academy of Chinese Medical Sciences, Beijing 100700, China; 3 National Resource Center for Chinese Materia Medica, China Academy of Chinese Medical Sciences, Beijing 100700, China

**Keywords:** cardiac aiging, whole-transcriptome sequencing analysis, ceRNA network, Yiqi Huoxue Yangyin (YHY) decoction

## Abstract

Background: Aging is a major factor for cardiovascular disease, and cardiac aging is closely related to the incidence of cardiovascular disease. Clarifying the mechanism of cardiac aging and finding reliable intervention is critical for preventing cardiovascular diseases and achieving healthy longevity. Traditional Chinese medicine Yiqi Huoxue Yangyin (YHY) decoction has unique advantage in the treatment of cardiovascular disease and aging. However, the associated molecular mechanisms remain unknown.

Purpose: The present study aimed to verify the efficacy of YHY decoction against cardiac aging in D-gal-induced mouse model, and explore the potential mechanism of YHY decoction treatment through whole-transcriptome sequencing technique, providing novel insights into the molecular basis of YHY decoction in treating cardiac aging.

Methods: The component of YHY decoction was identified by High Performance Liquid Chromatography (HPLC). D-gal-induced aging mouse model was established for this study. HE and Masson staining were applied to determine pathological changes of heart; telomere length, telomerase activity, AGEs and p53 were used to evaluate the degree of heart aging. Transcriptome sequencing, GO, KEGG, GSEA and ceRNA network were applied to analyze the potential mechanism of YHY decoction treatment of cardiac aging.

Results: In this study, we found that YHY decoction not only improved the pathological structure of aging heart, but also regulated the expression of aging-related markers, telomere length, telomerase activity, AGEs and p53, the myocardial tissue, suggesting that it has a specific effect in delaying cardiac aging. Whole-transcriptome sequencing showed that the total of 433 mRNAs, 284 lncRNAs, 62 miRNAs, and 39 circRNAs were significantly differentially expressed after YHY decoction treatment. According to the analysis results of KEGG and GSEA, the differentially expressed mRNAs were found significantly involved in immune system, cytokine-cytokine receptor interaction and cell adhesion molecules. The ceRNA network showed that miR-770, miR-324, and miR-365 are localized in center, mainly affecting the immune system, PI3K-Akt signaling pathway, and MAPK signaling pathway.

Conclusion: In conclusion, our results evaluated the ceRNA network of YHY decoction in treating cardiac aging for the first time, which could provide better understanding of the potential mechanism of YHY decoction treatment of cardiac aging.

## INTRODUCTION

The global population is aging rapidly. By 2050, one in six people in the world will be over age 65 (16%), up from one in 11 in 2019 (9%) [1]. Degenerative diseases caused by aging such as cardiovascular disease, osteoporosis, Alzheimer's disease, etc. are becoming major social challenges [2]. It is well known that aging is the major factor for cardiovascular disease [3], and the incidence of cardiovascular disease increases exponentially with aging. Cardiac aging is closely related to cardiovascular disease which accounts for more than 40% of deaths in age from 65 to 74, and 60% of deaths in people over 85 [4, 5]. Cardiac aging is characterized by damaged genomic DNA, shortened telomere length, altered epigenetic modifications, as well as accumulated senescent cells [6]. The structure and function of cardiomyocytes would be impaired with age, including pathological myocardial remodeling, left ventricular systolic and diastolic dysfunction, cardiac hypertrophy, and heart failure (HF) [7]. These changes increase heart vulnerability to stress and obviously increase the risk of cardiovascular disease. Therefore, it seems plausible to concentrate exploring the underlying mechanism of cardiac aging and improving the development of cardiac aging.

Traditional Chinese medicine Yiqi Huoxue Yangyin (YHY) decoction is composed of *Panax ginseng C.A.Mey.*, *Panax notoginseng*, *Rhizoma Chuanxiong* and *Dendrobium officinale Kimura et Migo*, which has unique advantages in the treatment of cardiovascular disease and aging [8–11]. Modern pharmacological studies indicated that ingredients in YHY decoction has effect of improving cardiac aging. *Ginsenoside Rg1* can effectively antagonize D-gal-induced myocardial tissue and ultrastructure damage through Keap1/Nrf2/ARE signaling pathway [12]. *Panax notoginseng* saponins can inhibit D-gal-induced aging of H9C2 cardiomyocytes, and the mechanism related to regulating mitochondrial function and oxidative stress [13]. In addition, *Dendrobii Caulis* can be used to combat age-related diseases such as Alzheimer's disease, hyperlipidemia, and diabetes [14]. However, due to the complexity of traditional Chinese medicine, the mechanism of YHY decoction has not be fully elucidated. Transcriptome refers to the sum of transcription products produced by cells, including coding RNA (mRNAs) and non-coding RNA (ncRNA, including lncRNAs, circRNAs, miRNAs, etc.). Transcriptome analysis can comprehensively reflect the molecular mechanisms of disease occurrence from the overall level, and its analysis strategy coincides with traditional Chinese medicine [15]. Moreover, an increasing number of ncRNAs have been found to be involved in the pathological process of aging and cardiovascular diseases.

Thus, the purpose of the current study is to verify the efficacy of YHY decoction against cardiac aging in D-gal-induced mouse model, and explore the potential mechanisms of YHY decoction treatment through whole-transcriptome sequencing technique. These results could provide novel insights into the molecular basis of YHY decoction in treating cardiac aging.

## MATERIALS AND METHODS

### YHY decoction preparation and analysis

YHY decoction consists of *Panax ginseng C.A.Mey.*, *Panax notoginseng*, *Rhizoma Chuanxiong* and *Dendrobium officinale Kimura et Migo* at a ratio of 2:3:4:4. *Panax ginseng C.A.Mey.*, *Panax notoginseng*, *Rhizoma Chuanxiong* were purchased from Beijing Tongrentang Co., Ltd., while *Dendrobium officinale Kimura et Migo* was purchased from Chishui Zhilv Jinchai Dendrobium Ecological Park Development Co., Ltd. *Panax notoginseng* was broken into coarse powder, together with *Panax ginseng C.A.Mey.* and *Rhizoma Chuanxiong*, and 70% ethanol was added to reflux for extraction twice. The ethanol was recovered and concentrated to a relative density of 1.20~1.30 (60° C), and then dried under reduced pressure. *Dendrobium officinale Kimura et Migo* was decocted twice with water and boiled twice, concentrated to 1.20~1.30 (60° C), and dried under reduced pressure. Above two dry powder were combined, crushed and mixed. According to the pharmacopoeia, the five main compounds (ferulic acid, notoginsenoside R1, ginsenoside Rg1, ginsenoside Re, and ginsenoside Rb1) of YHY decoction were analyzed.

### Animals and experiment designs

Male C57BL/6N mice were purchased from Beijing Vital River Laboratory Animal Technology Co., Ltd (license number: SCXK (Beijing) 2016-0006). Mice were housing in a room at 22° C with a 12-h light/dark circle and free access to food and water. Cardiac aging was induced by subcutaneous injection of D-gal dissolved in normal saline at a dose of 180 mg/kg body weight for 12 weeks. Mice were randomly divided into three groups: Control group (CH) were injected normal saline and given distilled water daily; Model group (MH) were injected D-gal and given distilled water daily; YHY decoction group (ZH) were injected D-gal and given 650 mg/kg YHY decoction daily. Mice were anesthetized and sacrificed after 12 weeks treatment.

### Histochemical and Masson staining

Heart tissue was fixed with 4% paraformaldehyde for 24 h, dehydrated, embedded in paraffin, and sectioned (4 μm), followed by Histochemical (HE) and Masson staining. For HE staining, the tissue slices were dewaxed with xylene I and xylene II, followed by dibenzene stepwise with descending gradient ethanol and rinsed with tap water. After nuclear staining, washing, differentiation and other experimental procedures, the slices were dehydrated, transparent, and mounted. For Masson staining, the dewaxed slices were placed in Masson mixture (Masson A and B=1:1) for 10 min, followed by 1% hydrochloric acid alcohol. The slices were treated by ponceau, phosphomolybdic acid, aniline blue and sealed with 1% glacial acetic acid. Finally, tissue sections were observed using an optical microscope (Olympus, Japan).

### Immunohistochemistry

After dewaxing the paraffin sections of the heart, they were placed in xylene and anhydrous ethanol reagents in turn. Next, the slices were subjected to antigen retrieval, and the treated slices were drawn in circles, and the primary antibody was added dropwise to the circles and incubated overnight. The second day continued to add secondary antibody, dehydrated after color development, and finally mounted the slide. Observation and analysis of the expression of AGE (1:200, 19003-1-AP, Proteintech) and P53 (1:200, 60283-2Ig, Proteintech) in myocardial tissue.

### Telomere length and telomerase activity analysis

All operations refer to the reagent instructions (GP1501, GP1502M, Gene poll, Beijing). Heart tissue was thoroughly ground in liquid nitrogen, and 1 ml of RLT was added to fully lysing. After adding chloroform, it was shaken and placed at room temperature for 2 min. Centrifuge at 12,000 rpm for 10 min, and transfer the upper aqueous phase to a new centrifuge tube. Add ethanol, transfer to Spin Column RM, centrifuge at 12,000 rpm for 20s, and discard the waste liquid in the Collection Tube. Add 350 μl Buffer RW1 to Spin Column RM, centrifuge at 10,000 rpm for 15 s, and discard the waste liquid. Put the Spin Column RM back in the 2 ml Collection Tube. Prepare DNase I mixture, add 80 μl DNase I mixture directly to the adsorption column, and incubate at 20-30° C for 15 min. Add 500μl Buffer RW2 to Spin Column RM (check whether absolute ethanol has been added before use), centrifuge at 10,000 rpm for 15 s, and discard the waste liquid. Put the Spin Column RM back in the 2 ml Collection Tube and repeat this process 1 time. Put the Spin Column RM back in the Collection Tube and centrifuge at 12,000 rpm for 1 min. Transfer Spin Column RM into a new 1.5 ml RNase-free Collection Tube, add 30 μl RNase-free Water, leave at room temperature for 5 min, and centrifuge at 12,000 rpm for 1 min. The cDNA obtained by reverse transcription was stored at -20° C. Relative quantitative analysis was performed using a real-time PCR instrument.

### Transcriptome sequencing and data analysis

Library construction and sequencing for mRNA, lncRNA, circRNA and miRNA expression were carried out by Biomarker Technologies Corporation (Beijing, China). Sequencing libraries were generated using NEBNext® Ultra™ Directional RNA Library Prep Kit for Illumina® (NEB, USA) according to the manufacturer’s recommendations and index codes were added to attribute sequences for each sample. PCR products were purified (AMPure XP system) and library quality was assessed on the Agilent Bioanalyzer 2100 and qPCR. Index-coded samples were clustered on acBot Cluster Generation System using TruSeq PE Cluster Kit v3/v4-cBot-HS (Illumina). After cluster generation, library preparation was sequenced on the Illumina Hiseq platform at Biomarker Technologies Corporation and paired-end reads were generated.

Differential expression analysis was performed using the DESeq R package (1.10.1). DESeq provide statistical routines for determining differential expression in digital gene expression data using a model based on the negative binomial distribution. The resulting P values were adjusted using the Benjamini and Hochberg’s approach for controlling the false discovery rate. mRNA/miRNA/lncRNA/circRNA with an adjusted P-value<0.05 and absolute value of log2 (Fold Change) ≥1.5 (miRNA absolute value of log2≥0.58) found by DESeq were assigned as differentially expressed.

Gene Ontology (GO) enrichment analysis of the mRNA differentially expressed genes (DEG mRNA) was implemented by the topGO R packages. KOBAS (Mao et al., 2005) software was applied to test the statistical enrichment of mRNA DEG in Kyoto Encyclopedia of Genes and Genomes (KEGG) pathways. The protein protein interaction (PPI) between DEG mRNA was analyzed by STRING database (http://string-db.org/) and was visualized in Cytoscape. The hub gene was filtered according to degree via the Cytohubba module in Cytoscape.

The Gene Set Enrichment Analysis (GSEA) analysis used the gene sets from MsigDB database, and broad GO and KEGG symbols were included. Log2 (Fold Change) of each differential group were scored as the background gene set to analyze the enrichment [16]. Finally, p value<0.001 and FDR<0.05 were controlled as significantly enriched gene sets. And pathways with corrected P-values of < 0.05 were considered significantly enriched, the visualized enrichment map of top 5 was performed by using clusterProfiler of R package.

The competing endogenous RNAs (ceRNA) network was constructed by first using targetscan or miranda database to predict DEG mRNA, lncRNA and circRNA-targeted miRNAs. Based on the relationship between miRNA-lncRNA, miRNA-circRNA, and miRNA-mRNA, the intersection miRNAs were found, and then with these miRNAs as the core, the co-expressed DEG lncRNA, DEG mRNA and DEG circRNA were screened for ceRNA network. Furthermore, Metascape online database was used to enrich and analyze the KEGG pathways of co-expressed mRNA in ceRNA network.

### Statistical analysis

All results were expressed as means±SE. Comparisons between two groups were calculated using Student’s t-test. For comparisons involving more than two groups, one-way analysis of variance (ANOVA) with a post hoc Bonferroni multiple comparison test was used to assess the difference. All statistical analyses were performed with Graphpad Prism 7.0 software. *P*<0.05 was considered statistically significant.

## RESULTS

### Identification of compounds in YHY decoction

**Figure 1:**
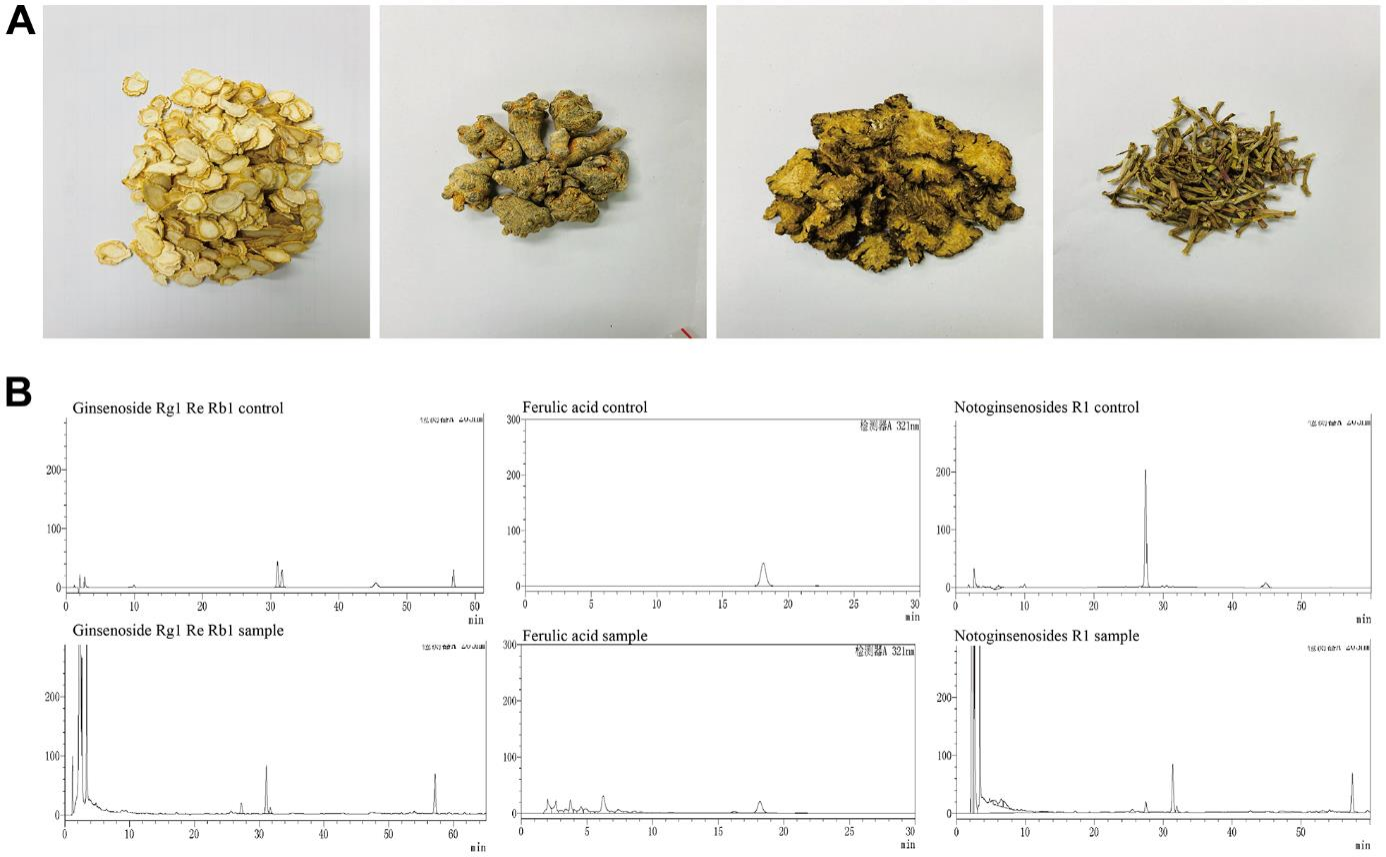
**Main compounds of the YHY decoction.** (**A**) Four traditional Chinese medicines of YHY decoction. (**B**) HPLC of ferulic acid, notoginsenoside R1, ginsenoside Rg1, ginsenoside Re, and ginsenoside Rb1 in YHY decoction.

High Performance Liquid Chromatography (HPLC) was employed to identify the main compounds in YHY decoction: ferulic acid, notoginsenoside R1, ginsenoside Rg1, ginsenoside Re, and ginsenoside Rb1, shown in Figure 1. Chromatographic analysis showed that the concentrations of ferulic acid, notoginsenoside R1, ginsenoside Rg1, ginsenoside Re, and ginsenoside Rb1 were 0.885 mg/g, 0.723 mg/g, 21.036 mg/g, 2.769 mg/g, and 20.003 mg/g, respectively.

### YHY decoction alleviated D-gal-induced cardiac aging

**Figure 2:**
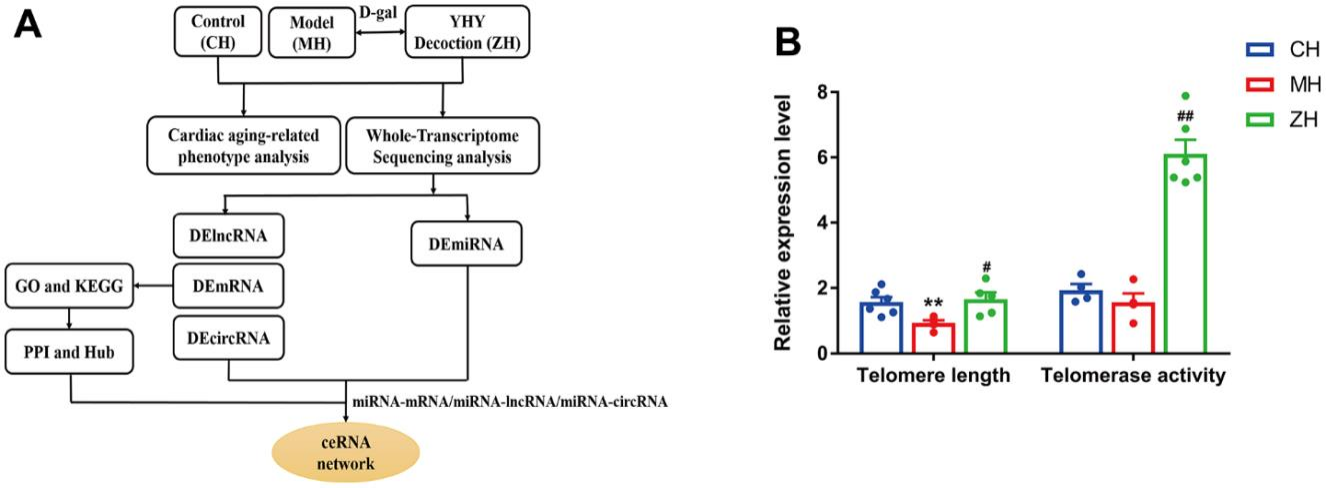
(**A**) The workflow of the study design. (**B**) Telomere length and Telomerase activity analysis (n=4-6). Compared with control group, *P<0.05, **P<0.01; compared with model group, ^#^*P*<0.05, ^##^*P*<0.01.

To evaluate the efficacy of YHY decoction on cardiac aging, we established D-gal-induced aging animal model as the reported, and explored the potential molecular mechanism of YHY decoction through whole-transcriptome sequencing technique (Figure 2A). Telomere length and telomerase activity are important indicators of detecting aging and senescence. Compared with the model group, telomere length and telomerase activity of the myocardium increased significantly after YHY decoction treatment (Figure 2B). Next, we used HE and Masson staining to detect the pathological morphology of mouse heart tissue.

**Figure 3:**
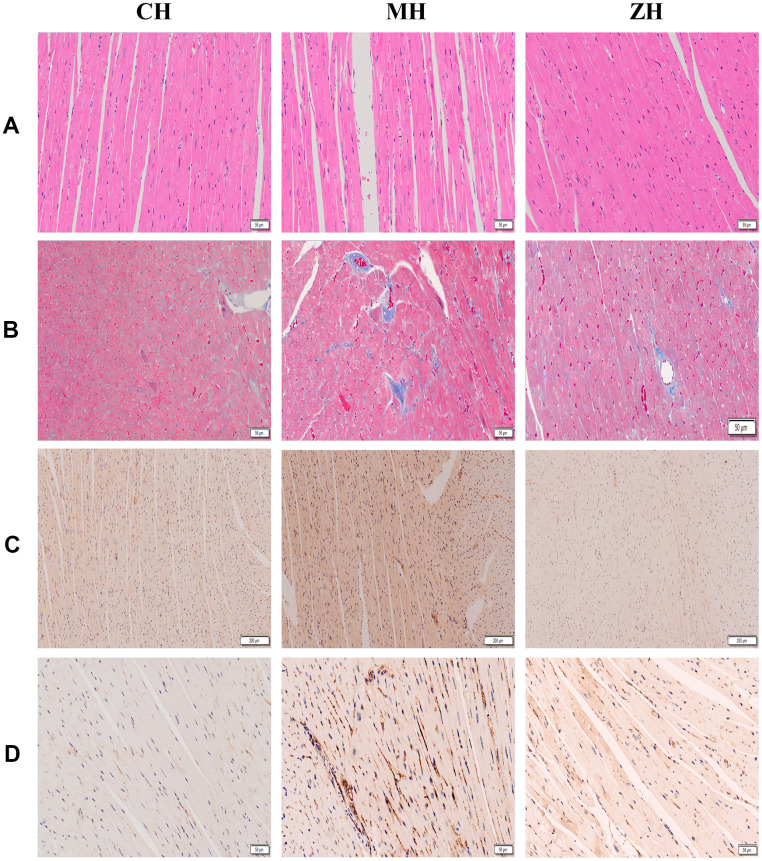
**Histopathological detection of mouse myocardium (n=3).** (**A**) HE staining; (**B**) Masson staining; (**C**) AGE protein immunohistochemical staining; (**D**) p53 protein immunohistochemical staining.

The results showed that YHY decoction can reduce myocardial fiber rupture, inhibit myocardial cell damage, reduce the content of collagen in myocardial tissue, and make myocardial cells arranged in a compact and orderly manner, suggesting that it can improve the cardiac pathological morphology of aging mice (Figure 3). In addition, the expression of aging markers AGEs and p53 was detected by immunohistochemistry and YHY decoction could reduce the content of AGEs and p53 in the myocardial tissue of aging mice, suggesting that it has a specific effect in delaying cardiac aging (Figure 3).

### Differential expression analysis of mRNA in YHY decoction treated myocardial tissue

**Figure 4:**
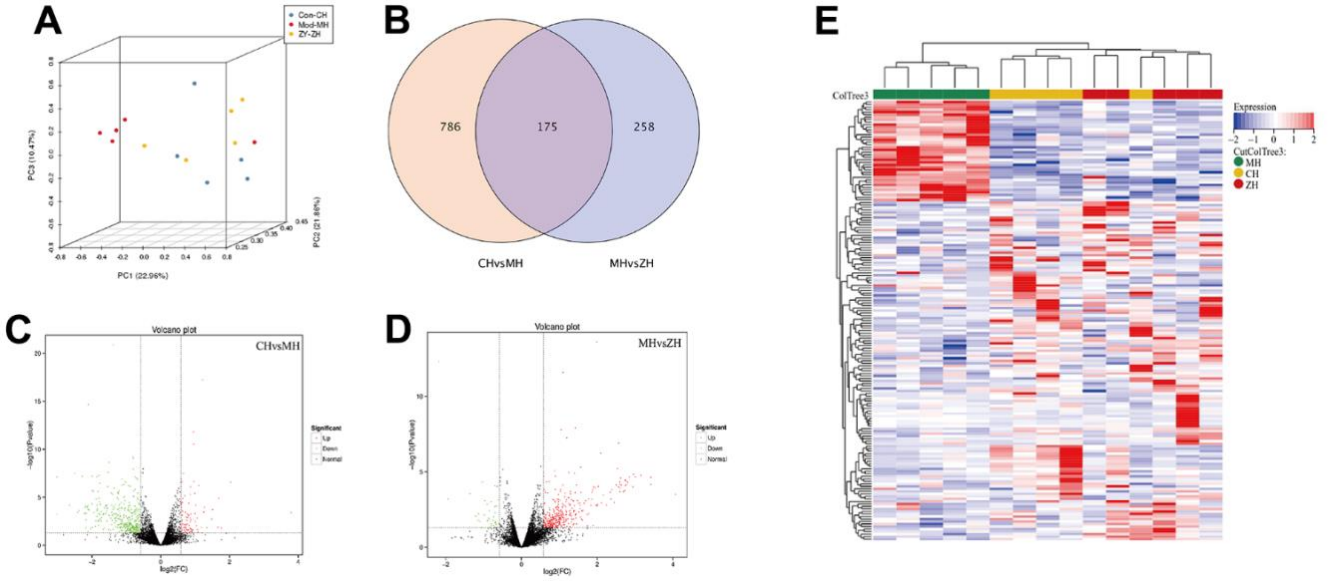
**The differentially expressed mRNA analysis of control group, model group and YHY decoction group (n=5).** (**A**) PCA diagram showing that samples distribution among different groups; (**B**) Venn diagram showing the DEGs of three groups; (**C**, **D**) Volcano plot of DEGs, screening parameters were P-value<0.05 and absolute value of log2 (Fold Change) ≥1.5; (**E**) Heatmaps of 175 DEGs intersected by three groups.

PCA analysis was performed on the mRNA expression levels of the three groups, and the results showed that the control group and the YHY decoction group had a clustering trend, which was significantly separated from the model group. According to the screening criteria, there are 961 differentially expressed mRNAs were identified between control group and model group, and there are 433 differentially mRNAs were identified between YHY decoction group and model group. The intersection of the three group has a total of 175 differential genes. The two-way clustering heatmap was showed in Figure 4E, from which we found YHY decoction treatment samples can be significantly separated from aging model group samples, indicating that the results of the differential expression analysis were reliable.

### Functional enrichment analysis of differentially expressed mRNAs

**Figure 5:**
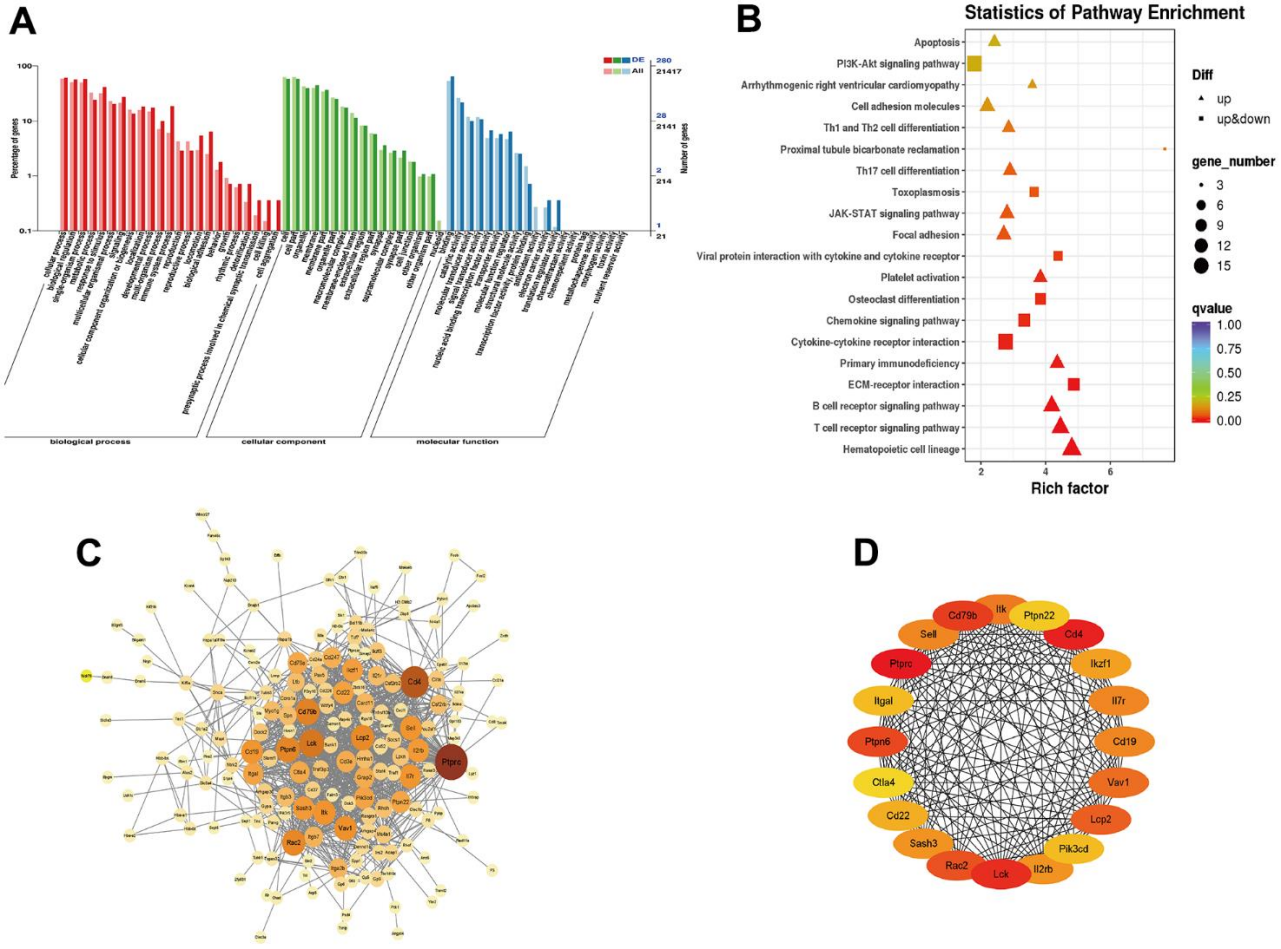
**Functional enrichment analysis of differentially expressed mRNAs.** (**A**) GO enrichment analysis of DEGs; (**B**) KEGG pathway analysis enrichment analysis of DEGs; (**C**) PPI network constructed using STRING online database; (**D**) Top 20 hub genes identified through degree method in cytoHubba.

To investigate the molecule mechanisms underlying the effect of YHY decoction against D-gal-induced cardiac aging, we subjected the differential expressed genes to GO and KEGG pathway analysis. GO pathway analysis showed that the differential genes mainly enriched in biological regulation, metabolic process, immune system process, membrane part, extracellular region part, et al. KEGG pathway analysis showed that differential genes were associated with Hematopoietic cell lineage, immune system (T and B cell receptor signaling pathway, primary immunodeficiency, Th17 cell differentiation, Th1 and Th2 signaling pathway), ECM-receptor interaction, platelet activation, cytokine-cytokine receptor interaction, cell adhesion molecules, JAK-STAT signaling pathway and PI3K-Akt signaling pathway, et al. (Figure 5A, 5B). The STRING online database was applied to construct the PPI network (Figure 5C), and analysis of hub genes was achieved by the degree method in cytoHubba (Figure 5D).

**Figure 6:**
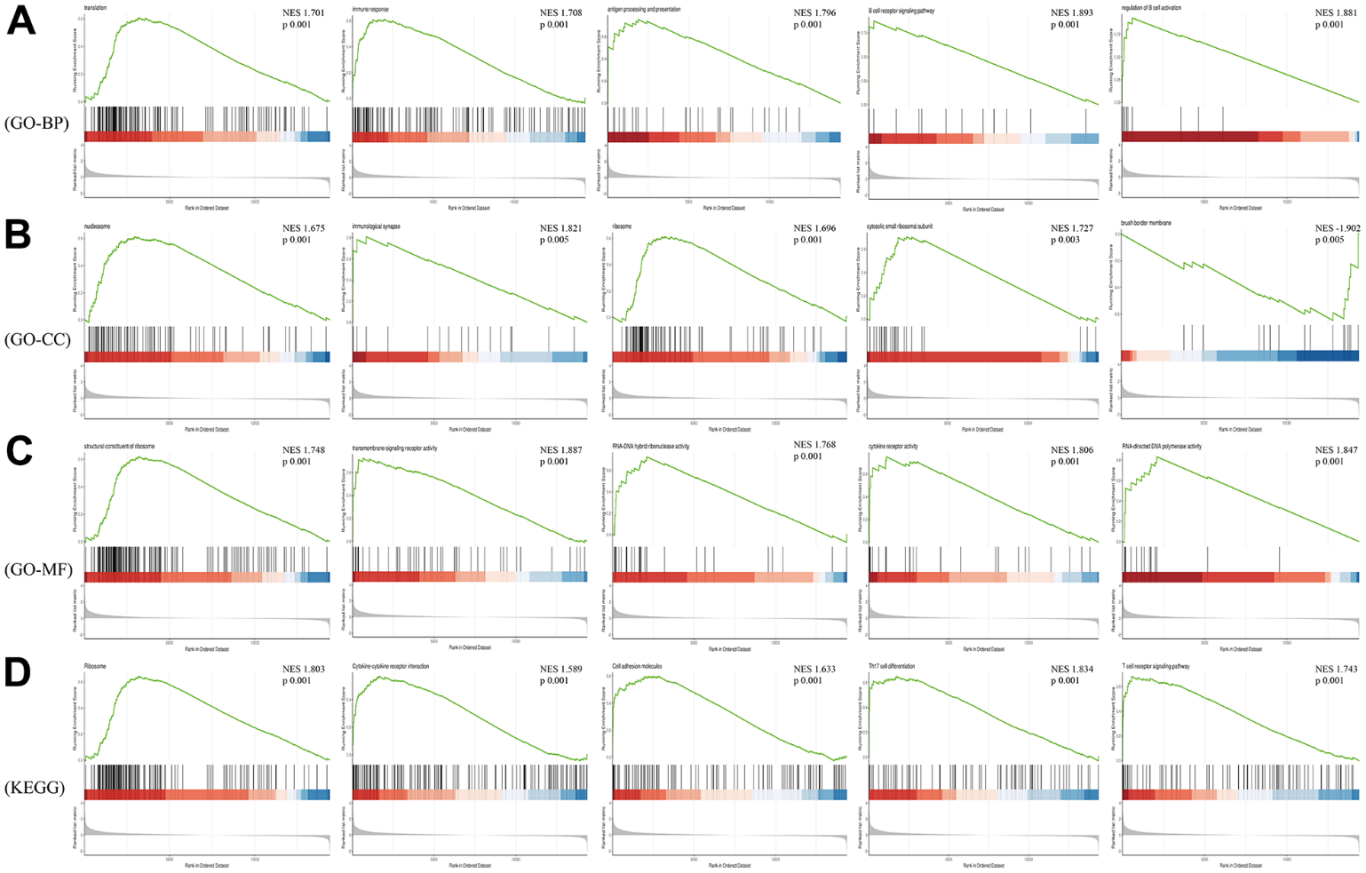
**GSEA analysis of differentially expressed mRNAs.** (**A**) Biological Process of GO; (**B**) Cellular Component of GO; (**C**) Molecular Function of GO; (**D**) KEGG pathway.

Considering that GO and KEGG Pathway analysis often focus on genes that are differentially expressed between two groups, it is easy to miss some genes that are not significantly differentially expressed but have important biological significance. Therefore, we employed GSEA analysis on the raw expression matrix of mRNAs. Translation, immune response (T and B cell receptor signaling pathway, Th17 cell differentiation), ribosome, cytokine-cytokine receptor interaction, cell adhesion molecules were the most significant enriched pathway (Figure 6).

### Identification of DEG lncRNAs, miRNAs and circRNAs in YHY decoction treated myocardial tissue

To further investigate the protective effect of YHY decoction treatment on the cardiac aging, the expression of lncRNA, miRNA and circRNA were also analyzed.

**Figure 7:**
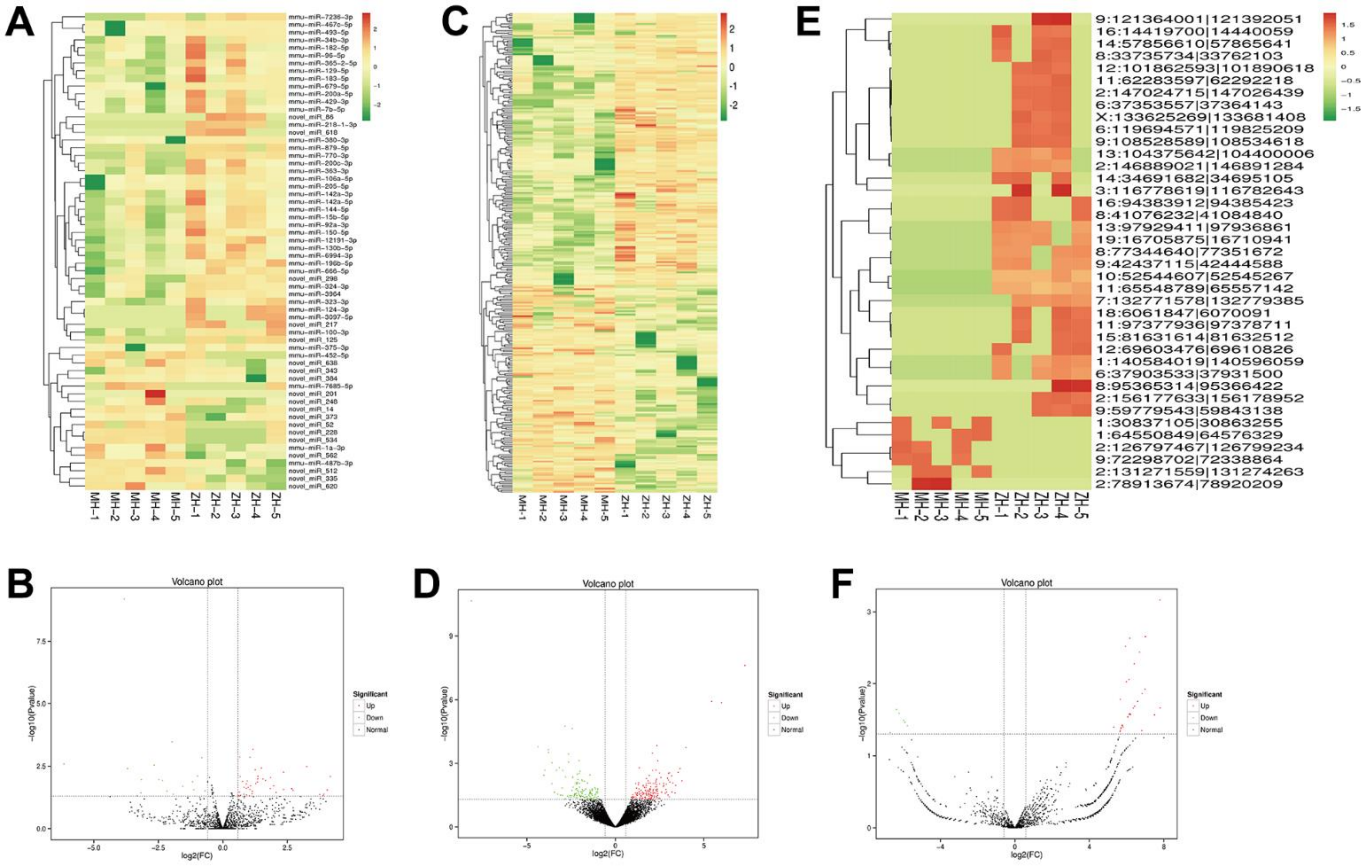
**Profiles of differentially expressed of miRNAs, lncRNAs and circRNAs between the model group and YHY decoction group.** Heatmap expression of DEG miRNAs (**A**), the volcano plot of DEG miRNAs (**B**), DEG lncRNAs (**C**), DEG lncRNAs (**D**), DEG circRNAs (**E**) and DEG circRNAs (**F**).

Heatmap clustering analysis showed that numbers of non-coding RNA were found to be differentially expressed between YHY decoction treated and untreated samples. We identified 284 lncRNAs, 62 miRNAs, and 39 circRNAs to be significantly differentially expressed (Figure 7).

### Construction of a potential lncRNA/circRNA-miRNA-mRNA ceRNA network

**Figure 8:**
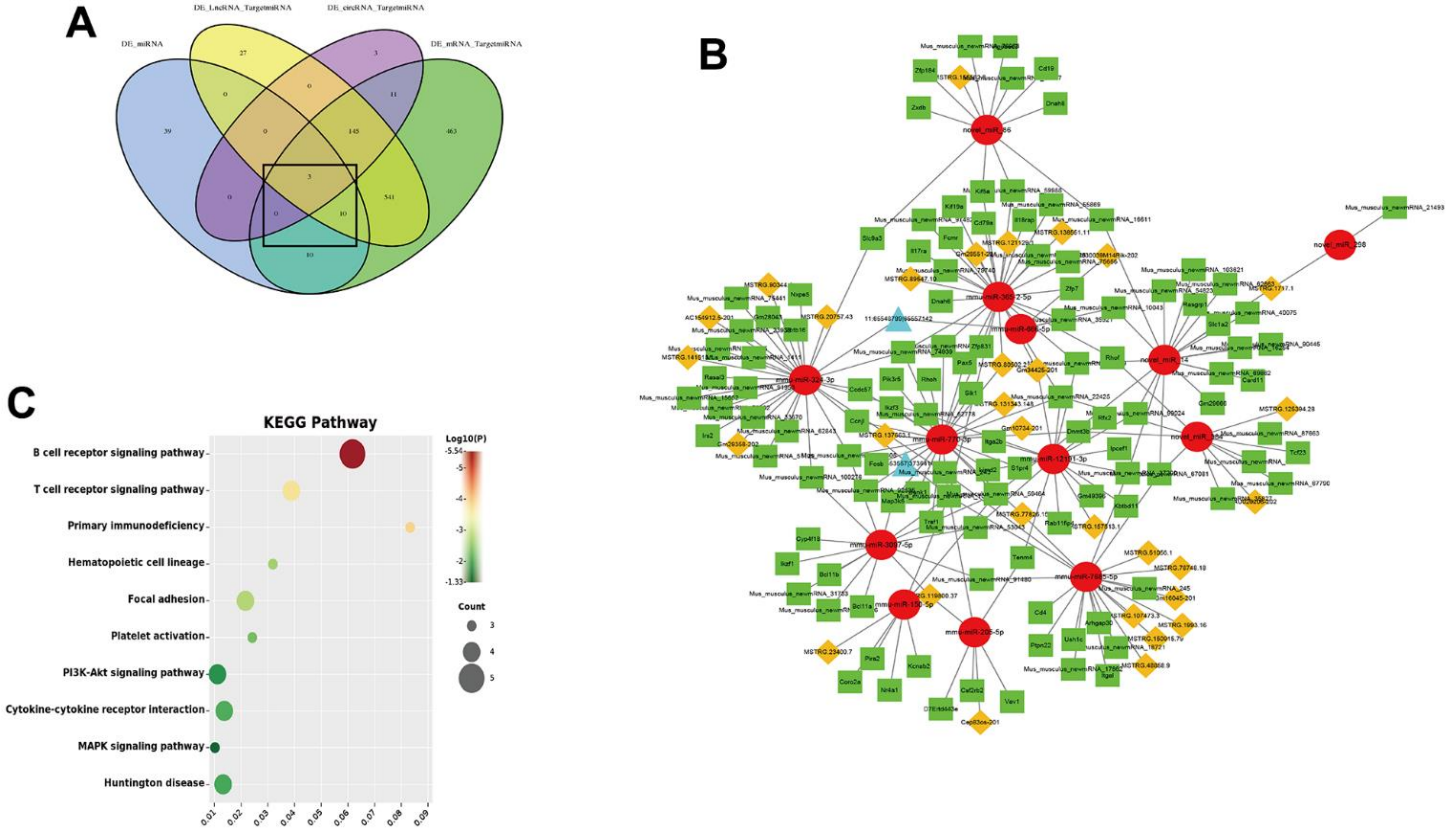
**CeRNA network of lncRNA/circRNA-miRNA-mRNA.** (**A**) Venn diagram of the interacting between mRNA-miRNA, lncRNA-miRNA and circRNA-miRNA; (**B**) ceRNA regulatory network (Red circle is miRNA, green squares is mRNA, yellow diamonds is lncRNA and blue triangles is circRNA); (**C**) KEGG pathway of co-expressed mRNA in ceRNA network.

The ceRNA hypothesis believes that endogenous RNA molecules can competitively bind to miRNAs, thereby indirectly regulating the expression of miRNA target genes. To obtain the competing relationships, we predicted the interacting between DEG mRNA-DEG miRNA, DEG lncRNA-DEG miRNA and DEG circRNA-DEG miRNA (Figure 8A). According to co-expression networks, we found that 13 DEG miRNA (miR-324-3p, miR-770-3p, miR-666-5p, miR-12191- 3p, miR-150-5p, miR-205-5p, miR-3097-5p, miR-365-2-5p, miR-7685-5p, novel miR-14, novel miR-298, novel miR-384 and novel miR-86) were core targets of the ceRNA network which could connected with 31 DEG lncRNA and 2 DEG circRNA. Furthermore, the 13 DEG miRNA could target 117 DEG mRNA (Figure 8B). The lncRNA/circRNA-miRNA-mRNA complex network was constructed by Cytoscape. KEGG pathway analysis showed that co-expressed mRNA in ceRNA network were associated with immune system (T and B cell receptor signaling pathway and primary immunodeficiency), Hematopoietic cell lineage, Focal adhesion, Platelet activation, PI3K-Akt signaling pathway, Cytokine-cytokine receptor interaction and MAPK signaling pathway (Figure 8C).

## DISCUSSION

Age is the most important determinant of cardiovascular health [17]. Biological aging disrupts cardiovascular system by promoting structural and functional changes in heart and vasculature, causing pathological changes such as left ventricular hypertrophy, decreased diastolic function, extracellular matrix remodeling, increased myocardial fibrosis, conduction block, etc. [18]. These changes eventually lead to increase the vulnerability of individuals to various cardiovascular diseases like coronary artery disease, hypertension, atherosclerosis, atrial fibrillation, and heart failure [19]. Cardiac aging begins with the senescence of the major constituent cells of the heart, such as cardiomyocytes, fibroblasts, endothelial cells and immune cells. With age, SA-β-gal activity, p21 and p16 protein expression in smooth muscle cells of the aortic root, atrial and ventricular myocytes increase, ultimately leading to cardiac aging and decreased cardiac function. Cardiac aging is a complex process often accompanied by telomere damage, oxidative stress, mitochondrial dysfunction, autophagy dysregulation, immunity imbalance and regulation of non-coding RNAs, etc. and intervention by any single pathway does not seem to be able to completely alleviate age-related cardiac dysfunction [20]. Therefore, clarifying the mechanism of cardiac aging and finding reliable intervention methods are critical for preventing cardiac aging and achieving healthy longevity.

Lots of researches have suggest that traditional Chinese medicine has good effects on cardiovascular aging, which plays an anti-cardiovascular aging role through mechanism such as anti-oxidation, inhibition of apoptosis, anti-inflammatory, and improving immunity, thereby preventing and treating cardiovascular diseases [21–24]. Hua et al. believes that traditional Chinese medicine can significantly improve the percentage of T-lymphocyte subsets and the activity of NK cells, thereby improving the immune function and disease resistance of the elderly [25]. Similarly, the study of BuShen HuoXue decoction found that it could attenuated hypertensive renal damage in aging SHRs, by significantly increasing Tregs and decreasing Th17 cells [26]. Ginsenoside Rb1 has been confirmed could inhibited vascular calcification and fibrosis and decreased the expression of cellular adhesion molecules, all of which subsequently ameliorated age -related vascular endothelium-dependent vasodilatation [27]. Polygonatum sibiricum is a biologically active Chinese herb that has widely applied to the therapy of cardiovascular diseases, which could suppress the lipid peroxidation and DNA damage of the heart in D-gal-induced aging mice via inhibiting oxidative stress [28]. D-gal-induced aging model is an ideal model for studying aging problems. This model uses large doses of D-gal to cause glucose metabolism disorders in animals, which in turn affects protein metabolism and lipid metabolism, resulting in a series of degenerative changes in tissues and cells [29, 30]. Based on this animal model, our findings indicated that YHY decoction could substantially increase telomere length and telomerase activity, reduce myocardial fiber rupture and the content of collagen, and inhibit the expression of aging markers AGEs and p53 in myocardial tissue of aging mice, suggesting that it has a specific effect in delaying cardiac aging.

YHY decoction has the advantages of multi-target, multi-effect, and multi-mechanism, but the specific mechanisms of anti-cardiac aging have not been elucidated. In this study, we first described the transcriptomic features of YHY decoction-treated D-gal-induced aging mouse. PCA, volcano plot, and heatmap analysis showed that the expression trend of control group and YHY decoction group was same, which proved that YHY decoction has a certain anti-aging effect on the heart caused by D-gal. According to the analysis results of KEGG, the differentially expressed mRNAs were found significantly enriched for Hematopoietic cell lineage, immune system, ECM-receptor interaction, platelet activation, cytokine- cytokine receptor interaction, cell adhesion molecules, JAK-STAT signaling pathway and PI3K-Akt signaling pathway, et al. Among these, immune system, cytokine-cytokine receptor interaction and cell adhesion molecules were also enriched by GSEA analysis. As research reports, aging is often accompanied by immune system dysfunction, including depletion of naïve T cells, degeneration of the thymus, decreased genetic polymorphisms of T cell receptors, accumulation of memory/effector T cells, and chronic inflammation, which is referred to as immunosenescence [31]. While both cytokine-cytokine receptor interaction and cell adhesion molecules are involved in the process of immune response and immune regulation. Accumulation of CD28^null^CD^4+^ and CD28^null^CD^8+^ T cells is the prominent changes during immunosenescence [32, 33]. These senescent T cells infiltrated myocardial tissue and produced amounts of pro-inflammatory cytokines and highly cytotoxic molecules, followed by age-related cardiac dysfunction. MMP9 is secreted by resident cardiac macrophages that increases with age, which in turn promotes cardiomyocyte hypertrophy and dysregulation of extracellular matrix homeostasis and collagen precipitation [34]. Research indicated that in addition to acting through antibodies and complement, activated B cells could also directly regulate cardiac function and induce cardiac remodeling by upregulating cytokines and chemokines [35]. Furthermore, JAK-STAT and PI3K-AKT are closely related to aging. Activation of JAK-STAT pathway can lead to secretion of SASP, occurrence of ROS-induced DNA damage, and inflammation of tissue microenvironment, which in turn leads to individual aging and aging-related diseases [36]. PI3K-Akt pathway acts an anti-aging role by regulating signal transduction and biological processes such as proliferation, apoptosis and autophagy [37, 38].

Next, the effects of YHY decoction on ncRNAs, including miRNAs, lncRNAs, and circRNAs, were evaluated in this study. Subsequently, the ceRNA network was constructed according to expression analysis of sequencing data of ncRNAs and the ceRNA hypothesis [39]. In recent years, the ceRNA hypothesis, as a new mechanism to explain the interaction between RNAs, has become a research hotspot for various diseases. As shown in our ceRNA network, miR-770, miR-324, and miR-365 were localized in the center, interacting with the most nodes, which indicated these miRNAs may be the key regulatory molecules for YHY decoction to exert anti-aging effect on the heart. Combined with the analysis of KEGG, it revealed that ceRNA network mainly exert influence on immune system, PI3K-Akt signaling pathway, and MAPK signaling pathway. For instance, miR-770 was constructed as the core in ceRNA network, while the upstream circRNAs, including 6:37353557|37364143, and lncRNAs, such as MSTRG.137663.1, MSTRG.77826.15, MSTRG.131343.148, MSTRG.80502.2, and Gm10734-201, acted as sponge to regulate Zfp831, Map3k6, Fosb, Pik3r5, Rhoh, et al. Previous studies have shown miR-770 is the potential candidates for investigating the aging process by controlling Wnt/chemokines and cytokines-related inflammation signaling pathways [40]. And miR-770 was also believed to play critical roles in the development and pathological mechanisms of dilated cardiomyopathy [41]. In our ceRNA network, Zfp831 and Rhoh, the downstream target of miR-770, have been identified to play critical roles in the differentiation of T follicular helper cells and T cells [42, 43]. Pik3r5 encodes the catalytic subunits of PI3K signaling pathway which could control AKT/NO/mTOR, NADPH oxidase, and TGF-b/Smad pathway that are involved in cardiovascular diseases [44, 45]. Additionally, Map3k6 constitutes a component of the MAPK pathway, chains of highly conserved serine/threonine protein kinases that communicate signals from cell surface receptors to the nucleus, influencing gene transcription [46]; MAPK signaling pathway participates in aging-induced changes in various organs including brain, liver, and heart [47]. The above two pathways, PI3K and MAPK signaling pathway, were both found to be regulated by miR-770, which is consistent with ceRNA network results in our study [48–50].

Despite these results, there are still some limitations in our study. The specific pathways for YHY decoction to prevent cardiac aging predicted by enrichment analysis has not been confirmed, and further research is required to elucidate its functions. Additionally, we have constructed a ceRNA network, but the mutual regulatory relationship between molecules has not been verified. And the heterogeneity caused by the small sample size may affect the reliability of the results. Finally, in this study, transcriptomic sequencing was performed on the whole heart tissue, ignoring the differences between cell types, such as cardiomyocytes, endothelial cells, fibroblasts and immune cells, so that single cell sequencing technology can be considered in further research.

## CONCLUSIONS

In conclusion, the function of YHY decoction on inhibiting D-gal-induced cardiac aging was demonstrated. After that, we identified DEG mRNAs, lncRNAs, circRNAs, and miRNAs in cardiac tissue of aging mouse exposed to YHY decoction and constructed a circ/lncRNA-miRNA-mRNA networks. In the ceRNA network, miR-770, miR-324, and miR-365 are localized in the center, mainly affecting the immune system, PI3K-Akt signaling pathway, and MAPK signaling pathway, and playing an anti-aging role on the heart. Our results evaluated the ceRNA network of YHY decoction in treating cardiac aging for the first time, which could provide a better understanding of the potential mechanisms of YHY decoction treatment of cardiac aging.
